# Small Molecules in Rare Tumors: Emerging Role of MicroRNAs in GIST

**DOI:** 10.3390/ijms19020397

**Published:** 2018-01-30

**Authors:** Juozas Kupcinskas

**Affiliations:** 1 Institute for Digestive Research, Academy of Medicine, Lithuanian University of Health Sciences, Eiveniu str. 2, LT-50009 Kaunas, Lithuania; juozas.kupcinskas@lsmuni.lt; Tel.: +370-37-326898; 2 Department of Gastroenterology, Academy of Medicine, Lithuanian University of Health Sciences, Eiveniu str. 2, LT-50009 Kaunas, Lithuania

**Keywords:** GIST, microRNA, isomiR, non-coding RNA, biomarker

## Abstract

Gastrointestinal stromal tumors (GISTs) are the most common mesenchymal tumors of gastrointestinal tract. GISTs have very different clinical phenotypes and underlying molecular characteristics that are not yet completely understood. microRNAs (miRNAs) have been shown to participate in carcinogenesis pathways through post-transcriptional regulation of gene expression in different tumors. Over the last years emerging evidence has highlighted the role of miRNAs in GISTs. This review provides an overview of original research papers that analyze miRNA deregulation patterns, functional role, diagnostic, therapeutic and prognostic implications in GIST as well as provides directions for further research in the field.

## 1. Introduction

Gastrointestinal stromal tumors (GISTs) are the most common mesenchymal tumors found in the gastrointestinal tract. The major driving elements in GIST pathogenesis are linked with mutations in tyrosine kinase family (KIT) or platelet-derived growth factor (PDGFRA) gene [1,2]. Only a small proportion of GISTs appear to be associated with neither KIT or PDGFR sporadic mutations and are referred to as wild-type (WT) tumors [3]. GISTs most commonly arise from the fourth layer of the gastrointestinaltract wall (muscularis propria), and less often from the more superficial muscularis mucosa layer [4]. These tumors develop from a particular cell type in the wall of the GI tract called the interstitial cells of Cajal [5]. GISTs may be encountered in any part of the gastrointestinal tract, but most frequently they are found in the stomach [4]. Patients with GIST may present with gastrointestinal bleeding, intestinal obstruction, abdominal mass or vague abdominal pain; however, in most cases the course of disease is asymptomatic [6]. Improved diagnostic modalities and newly available treatment agents improved survival of GIST patients over the last decade; however, the prognosis of advanced diseases still remains poor [7]. The gold standard therapy for advanced disease is imatinib that offers a good and stable response for approximately 2–3 years, but resistance in long term treatment is very common [8]. Sunitinib and regorafenib are the therapeutic options of imatinib-resistant GIST; however their efficacy is also limited in progressive disease cases [9]. Therefore, further fundamental and clinical studies are being conducted in order to identify underlying molecular pathways in GIST in order to provide novel therapeutic options. 

MicroRNAs (miRNAs) are small non-coding RNAs involved in post-transcriptional regulation of gene expression [10]. Over the last years tremendous research efforts have been taken to identify the role of miRNAs in carcinogenesis [10]. To date, deregulation of miRNAs has been identified in virtually all cancers including gastric [11,12], colorectal [13], pancreatic [14], pediatric gliomas [15] and many others [16,17]. A number of different approaches have been used to analyze miRNAs mediated cancerogenic effects including miRNA profiling studies [18,19,20,21], analysis of polymorphisms in the genes encoding miRNAs [22,23], analysis polymorphism of miRNA targeted genes [24], estimation of methylation of miRNA related gene promoter regions [11] or functional role in cell lines and different molecular pathways [16,25]. Significant alterations at fundamental molecular level led to further studies providing important diagnostic and prognostic implications of miRNAs as valuable biomarkers in cancer [11,26,27]. Furthermore, a series of clinical trials have been and are being conducted that include different miRNA molecules containing treatment regiments [28,29].

An increasing number of studies have been performed trying to identify the role of miRNAs in GIST. The first study was conducted back in 2008 by Subramanian et al. , which clearly showed that miRNA expression profiles are distinct among different sarcoma types [30]. Later profiling studies have identified unique miRNA signatures related to underlying mutations [31,32,33,34], anatomical site [32,35], malignant potential [32,35,36], or prognosis [35] and even treatment [32,37]. Next to profiling studies, a number of very important functional studies have been performed that identified crucial molecular pathways targeted by miRNAs including KIT/AKT, PDGFRA genes [37,38,39]. Several miRNAs have been identified as potential prognostic biomarkers [35,40,41] while others have been associated with imatinib resistance [42,43,44]. Increasing awareness on the role of miRNAs in GIST paves the way for further studies in the field that could advance clinical practice.

This review provides an overview of all original research papers that analyze miRNA deregulation patterns, functional role, diagnostic, therapeutic and prognostic implications in GIST as well as provides directions for further research in the field. 

## 2. Methods

**Figure 1:**
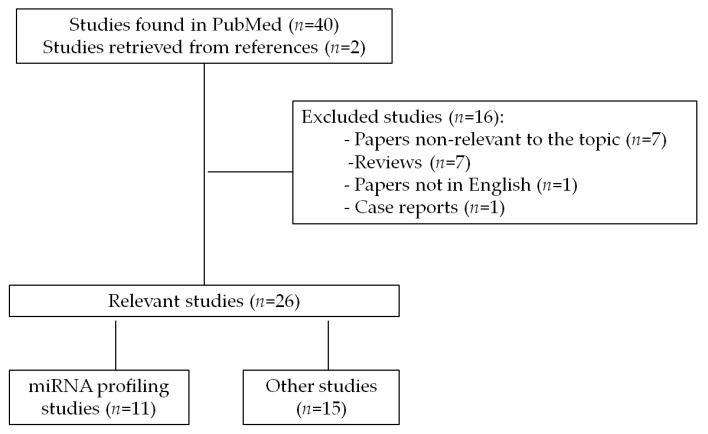
Flow diagram of the original research papers search strategy and identification of studies relevant for review.

A comprehensive search was carried out in order to identify all papers related to the role of miRNAs in GIST published from January 2000 to January 2018. The review included all studies published in PubMed database that were retrieved under the search terms “GIST and microRNA”. The search revealed 40 papers and two papers were additionally included manually based on citations in related publications. Out of 42 papers 26 relevant original research publications were included in the current review (seven papers were omitted as non-relevant to the topic, seven papers were reviews, one case report and one paper not in English). The literature search strategy and selection process is shown in flow diagram (Figure 1).

## 3. miRNA Expression Profiles in GIST

An excellent previous review by Nannini et al. has systemically analyzed miRNA profiling studies in GISTs that were published up to the year 2016 [21]. Since then several other papers came out and to date there are more than 10 studies that have looked at miRNA expression deregulation patterns in GIST. The major aspects of these studies are summarized in Table 1. It must be pointed out that most of the studies included relatively small numbers of tumor samples ranging from 3 to 70 (Table 1). It is very difficult to compare the results of these different profiling studies in between because most of them used very different comparison strategies in terms of GIST location, malignant potential, metastasis, adult vs. pediatric, wild-type vs. mutated, GIST vs. other sarcomas, etc. (Table 1). One of major problems in designing GIST miRNA profiling studies is related to the absence of ideal control tissue that could be used as a reference. Furthermore, the techniques of miRNA profiling in these studies vary a lot: most of the studies used different microarray techniques, while one study employed real time-polymerase chain reaction (RT-PCR) and only one other study was based on next-generation sequencing approach which is considered the most informative in miRNA profiling studies (Table 1). Nevertheless, all of these studies have revealed very important miRNA related deregulation patterns in GIST as many of deregulated molecules were further explored in functional GIST studies.

The first paper that looked at miRNA expression profiles in GIST tumors was published in 2008 by Subramanian et al. [30]. They looked at miRNA profile comparing different types of sarcoma and have clearly shown that miRNA expression profiles are distinct among the sarcoma types [30]. A study by Choi et al. 2010 determined that miRNA expression patterns of GISTs were closely related to the status of 14q loss, anatomic site, and tumor risk [31]. The importance of GIST tumor location for miRNA profile was also shown by a German study [32]. Studies looking at miRNA profile in GIST tumors baring different mutation profiles (WT vs. KIT vs. PDGFRA) have elegantly shown that expression of miRNA is highly dependent on mutation status [45]. This observation was also supported by a study of Bachet et al. that showed that patterns of miRNA expression tumors and cell lines depend on heterozygous/hemizygous status of KIT mutations with each mutation of KIT driving specific oncogenic pathways [46]. MiRNAs appear to be novel biomarkers to distinguish malignant from benign GISTs, which may be helpful to understand the mechanisms of GIST oncogenesis [36]. Distinct miRNA expression patterns have also been identified in relation to imatinib response and metastasis [35]. Kelly et al. have shown that miRNA profiles segregate pediatric and adult GIST tumors [33] indicating that underlying pathophysiological molecular pathways might be differently affected at different ages of disease development. The only study that used high-throughput miRNA profiling of 15 paired GIST and adjacent normal tissue samples was performed using small RNA-seq approach and overall analyzed 1672 known miRNAs [47]. This study found miR-509 up-regulation in epithelioid and mixed cell types as compared to spindle type, while miR-215 showed negative correlation with risk grade of GIST. The latter study is also the only one which looked not only at differentially expressed miRNAs but also identified differentially expressed isoforms of miRNAs [47]. 

Taking into account the heterogeneity of published profiling studies it is difficult to identify miRNAs that would relate specifically to GIST. Nevertheless, certain miRNAs have been identified as repeatedly deregulated in variable clinical and molecular context: miR-214 [33,35], miR-210 [33,35], miR-23b [31,35], miR-221/miR-222 in [30,48], let-7 family [30,33,35]. Aforementioned miRNAs have been also identified as important carcinogenesis mediators in other gastrointestinal cancers [16,49]. Overall current miRNA profiling studies clearly show that expression patterns of these molecules are highly dependent on tumor subtypes, underlying mutations, morphological features and clinical behavior; therefore, larger scale studies with very well phenotypically described samples are needed.

## 4. Functional Role of miRNAs in GIST

**Figure 2:**
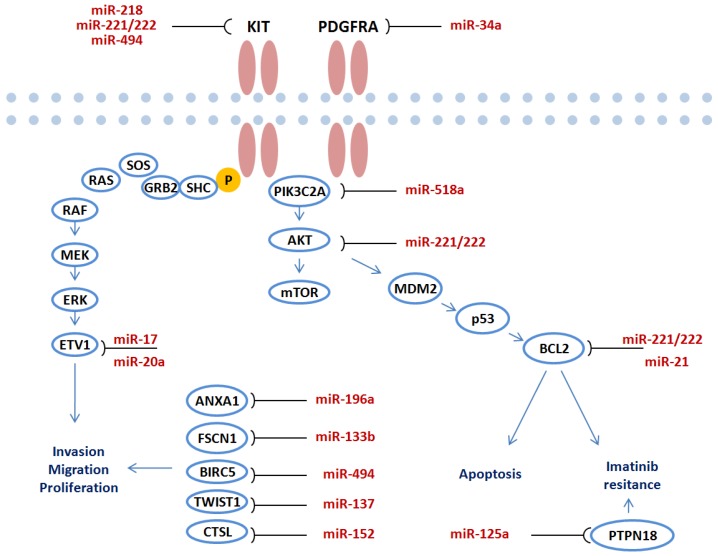
Functional role of deregulated miRNAs in GIST carcinogenesis. miRNAs regulate target gene expression and mediate invasion, migration, proliferation, apoptosis and imatinib resistance through key molecular pathways.

One of the biggest challenges in order to elucidate the role of miRNAs in carcinogenesis stems from functional studies in different malignancies [16,18]. Over the last decade a number of important functional analyses have been carried out to verify the target genes of miRNA in GIST. The overview of miRNAs and identified target genes in GIST is presented in Table 2. A large number of performed studies show that miRNAs directly target crucial genes in GIST pathogenesis including KIT/AKT, PDGFRA pathways. Deregulated miRNAs regulate target gene expression and mediate invasion, migration, proliferation, apoptosis or imatinib resistance through key molecular pathways (Figure 2).

### 4.1. The Functional Role of miR-221/miR-222

MiR-221 and miR-222 are two highly homologous miRNAs and their deregulation has been identified in different cancers [56]. MiR-221/222 may function both as oncogenes or tumor suppressors, depending on tumor biology [56]. Taking into account the deregulation of miR-221/miR-222 in GIST profiling studies a number of studies tried to evaluate the functional role of these two miRNAs in tumor development (Table 2; Figure 2). Koeltz et al. were the first to show that miR-221 and miR-222 can act as regulators of KIT expression in GISTs [53]. They showed that expression levels of miR-221 and miR-222 were significantly repressed in KIT-positive GISTs, compared to normal tissue, whereas KIT-negative GISTs exhibited a completely inverse expression pattern. Another German study has shown that miR-221 and miR-222 are downregulated in wild-type and mutated GISTs; furthermore, they induce apoptosis in vitro by a signaling cascade involving KIT, AKT and BCL2 suggesting that overexpression of these miRNAs seems to functionally counteract oncogenic signaling pathways in GIST [39]. MiR-221/222 were also significantly lower expressed in GIST vs leiomyosarcomas and normal gastrointestinal control tissues while over expression of miR-222 in GIST cell lines severely inhibited cell proliferation through targeting KIT [48]. The study also showed that MiR-17/20a directly targeted ETV1 and affected apoptosis and cell cycle (Table 2).

### 4.2. The Functional Role of miR-494

MiR-494 promotes cell proliferation, migration and invasion in hepatocellular carcinoma [57], suppresses tumor growth of epithelial ovarian carcinoma [58] and regulates other important cellular functions in cancer [59]. Two studies have looked at the functional role of miR-494 in GIST and have identified two very important direct targets for this miRNA (Table 2). The first study by Kim WK et al. [55] showed that miR-494 is a negative regulator of KIT in GISTs and overexpression miR-494 in GISTs may be a promising approach to GIST treatment. The crucial KIT pathway was also affected by miR-494 through targeting baculoviral inhibitor of apoptosis repeat-containing 5 (BIRC5). This study showed that miR-494 suppressed GIST proliferation [54]. 

### 4.3. The Functional Role of Other miRNAs

A growing number of studies have demonstrated that miR-152 may act as a tumor suppressor gene by regulating target genes, which are associated with cell proliferation, migration and invasion in human cancers [60]. The key findings by Lu et al. study provided evidence suggesting that in GIST miR-152 functions by means of binding to cathepsin L to induce cell apoptosis and inhibit proliferation, migration, and invasion [52]. Another important miRNA-miR-137 has been implicated in different cancers including gastric [11] and colorectal [13]; but it also appears to regulate epithelial-mesenchymal transition in gastrointestinal stromal tumor via TWIST1 downregulation [51]. The other research group found that miR-34a downregulated a number of predicted target genes, including PDGFRA. RNA interference-mediated knockdown of PDGFRA in GIST-T1 cells suppressed cell proliferation, suggesting the tumor suppressive effect of miR-34a is mediated, at least in part, through targeting PDGFRA [37]. The same miRNA was also identified to be associated with other cancers [61]. A Japanese study looking at miR-196a and head-tail axis transcript antisense RNA gene (HOTAIR) in high-risk gastrointestinal stromal tumors found that ANXA1 was a direct target of miR-196a and affected invasion of tumor cells [34].

### 4.4. Proposed Functional Role of miRNAs by Bioinformatical Analyses

One of the major approaches to identify potential target genes of miRNAs is through bioinformatic analyses mapping miRNA sequencing against target gene sequences [62]. It is known that mRNAs of different genes may contain many or sometimes even extraordinarily large numbers of miRNA binding sites [62]. Among the targets that correlated with the miRNA expression, one study was able to identify 17 mRNA–miRNA networks in GIST [45]. Using the bioinformatical target gene identification approach they further showed that miR-139-5p, miR-455 and let-7b may regulate the the insulin-like growth factor I receptor (IGF1R) while cyclin dependent kinase 6 (CDK6) may be modulated by miR-139-5p/let-7b [45]. A very comprehensive bioinformatical analysis of miRNAs associated pathways and potential target genes of miRNAs in GIST was presented by Gyvyte et al. [47]. This study showed that cytokine-cytokine receptor interaction, erythroblastic leukemia viral oncogene (ERBB) signaling, p53 signaling, mitogen-activated protein kinase (MAPK) signaling, cell cycle, mTOR signaling, janus kinase/signal transducer and activator of transcription (JAK/STAT) signaling pathways were significantly affected in GIST tumors. Further enrichment analysis underlying possible functions of enriched miRNAs, predicted and validated targets were retrieved and mapped to the above mentioned deregulated pathways. It must be pointed out that all predicted target genes of miRNAs by this approach must be further validated in vitro studies using GIST cell lines and appropriate molecular techniques.

## 5. Potential Role of miRNA in GIST Treatment

One of the most important aspects of miRNAs is their potential application in cancer treatment. Anticancer therapies based on miRNAs are currently being developed with a goal to improve outcomes of cancer treatment [29]. Different miRNA related treatment strategies are being investigated including inhibition of miRNA, miRNA sponges, anti-miRNA oligonucleotides, small molecular inhibitors of specific miRNAs or replacement of miRNA [28]. Currently, there are several miRNAs that are in clinical trials, some already in phase II stage [28]. Additionally, various molecular biomarkers are in attempt to be identified that could guide personalized management of GIST patients [8,63].

The potential role of miRNA with respect to potential application in GIST has been explored in a series of fundamental studies. Several studies tried to address a very important clinical challenge in GIST treatment—imatinib resistance. miRNA-21 mimic transfection markedly aggravated the imatinib-mediated growth inhibition and apoptosis induction in GIST-T1 cells [50]. Interestingly, miR-21 was also found to be induced by Epstein-Barr virus in multiple myeloma cell lines [64]. In addition, low expression of miR-518a-5p upregulated PIK3C2A and impaired cellular response to the drug, causing resistance to imatinib was observed in GISTs [43]. In parallel, Fan R et al. showed that miR-218 over-expression can improve the sensitivity of GIST cells to imatinib mesylate [42]. A study by Durso et al. showed that modified miRNAs 221/222 with altered nucleotides in the seed region are effective inhibitors of KIT gene expression and may help to overcome drug resistance concerns [44]. One study suggested that miR-218 loaded nanoparticle could act as a tumor suppressor miRNA in the treatment of GIST [65]. It must be pointed out that to date none of miRNAs have been tested in GIST clinical trials and their actual use in treatment algorithms is awaiting establishment.

## 6. Prognostic Role of miRNAs in GIST

Multiple miRNAs have been suggested as prognostic biomarkers in different malignancies including gastric cancer [11], non-Hodgkin lymphoma [66], hepatocellular carcinoma [67] and others. Different subtypes GISTs have different risk assessments with respect to the disease recurrence or metastasis, age of occurrence, depending on their location, size, and number of mitosis [68]. Several studies looking at miRNAs also tried to identify their prognostic role in GIST patients. Yamamoto et al. 2013 showed that fascin-1 might is a direct target of miR-133b; furthermore, they showed that fascin-1 overexpression was significantly correlated with shorter disease-free survival time and aggressive disease behavior including tumor size, mitotic counts, risk grade, blood vessel invasion and mucosal ulceration [40]. Overexpression of miR-196a in GIST tissues was associated with high-risk grade, metastasis and poor survival [34]. The same group also showed that low miR-186 expression is associated with metastatic recurrence and a poor prognosis [41]. Similarly miR-1915 expression correlated with disease-free survival and overall survival in GIST patients [35]. These preliminary results suggest that miRNAs, most likely in combination with other biomarkers, might be a useful prognostic biomarker for stratifying patients in terms of their prognosis.

## 7. Future Perspectives

### 7.1. Identification of isomiR Profiles and Their Functional Role in GIST

Advances of next generation sequencing techniques revealed that miRNAs can differ from archetype sequences listed in publically available databases and may vary both in length of the molecule and its’ sequence [69,70]. The varying miRNA sequences have been named isoforms of miRNAs or isomiRs. To date, our knowledge about these molecules in cancer still remains very limited. Overall, studies clearly show that archetype miRNAs cover only a small fraction of all miRNA molecules [71] suggesting that many cancer related effects might be missed looking only at the standard miRNAs. So far isomiRs have been studied only in few cancers including breast cancer, melanoma and gastric cancer [71,72,73], but the number of these studies is expanding every year. It must be pointed out that in 2017 a first study looking at the role isomiR has also been published on GISTs revealing different expression profiles of these molecules in GIST tumor and tumor adjacent tissue [47]. This study identified 219 deregulated isomiRs in 89 unique miRNA sequences between GIST and tumor adjacent tissues. The function of these miRNAs isoforms appears to be very important. For example miR-222 which is deregulated in GIST [53] has been shown to have several isoforms with different functions: a study demonstrated that 3′ end heterogeneity of miRNA sequences has dramatic implications for the phenotype of miR-222 transfected cells [74]. The functional role of the isoforms of miRNAs has not been studied in GIST and further studies must be carried out in order to elucidate potentially very important role of these molecules.

### 7.2. Circulating miRNAs in Blood of GIST Patients 

MiRNAs that were detected in blood were described as circulating miRNAs [75]. To date, there is no study looking at the role of circulating miRNAs in GIST patients. This would be extremely important in patients with an early disease when symptoms are usually absent—in this context they may serve as molecular tools for disease screening purposes. Several studies have shown that circulating miRNAs may be used as very useful biomarker in different malignancies including gastric [18], colorectal [76], pancreatic [77] and other cancers. Circulating miRNA profiles have been also analyzed in patients with advanced cancer suggesting their potential role to identify metastasis [78]. It worth pointing out that circulating miRNA-92b-3p has been identified as a novel biomarker for monitoring of sarcoma [79]; therefore, analysis of circulating miRNAs in blood must be considered in further studies.

### 7.3. Single Cell Profiles of miRNAs in GIST

Until now numerous studies have shown that circulating miRNAs may serve as diagnostic and prognostic biomarkers for different malignancies; however, the origin of these molecules in the blood until recent years was completely not clear. Some high quality papers have shown the potential to exploit highly sensitive methods to detect mRNA and miRNA in isolated human cell types using flow citometry [80] or microfluidic-based method which enables the detection of miRNAs in single intact cells by flow- fluorescence in situ hybridization (FISH) [81]. A recent study by Juzenas et al. clearly showed that miRNAs are differentially expressed in individual blood cell groups [82]. They identified blood cell-specific miRNA and isomiR expression patterns and created a complete miRNA catalogue of human peripheral blood, which might be used as a reference for future studies in the field. All the studies conducted on GIST and miRNAs do not look at the effects of these molecules at a single cell level; therefore, further profiling studies employing flow citometry, microfluidic systems or other more precise single cell oriented techniques are needed. This may allow to identify more specific GIST related miRNA profiles and deeper insights in the pathogenesis of this malignancy.

## 8. Conclusions

Over the last years emerging evidence has elucidated the role of miRNAs in GIST. Increasing number of studies revealed miRNA deregulation patterns in different disease associated phenotypes and identified the functional role of numerous miRNAs. miR-23b, miR-221, miR-222, miR-210, miR-214 were most frequently reported as deregulated miRNAs in various GIST phenotypes. MiR-34a, miR-218, miR-221/222, miR-494 and other important miRNAs target crucial KIT/PDGFRA pathways and affect proliferation, migration and invasion processes in GIST. Furthermore, miR-21, miR-221/222, miR-125a are implicated in complex imatinib resistance mechanisms. A number of miRNAs including miR-186, miR-196a, miR-133b also appear to have important diagnostic, therapeutic and prognostic implications for GIST patients. Nevertheless, further high quality clinical and fundamental studies are needed in order to translate current observations into clinical practice. In order to achieve these goals novel methodological miRNA related study approaches are needed including next generation sequencing, single-cell analyses, high quality functional studies, etc.

## Figures and Tables

**Table 1:** Studies on miRNA expression profiles in gastrointestinal stromal tumors (GIST).

Study	miRNA Profiling Method	Number of Investigated miRNAs	Compared Groups (*n*)	Number of Upregulated miRNAs	Number of Down-Regulated miRNAs
Subramanian et al., 2008 [30]	microarray	328	GIST (*n*, 8) vs. sarcomas (*n*, 25)	15	9
Choi et al., 2010 [31]	microarray	470	Gastric (*n*, 15) vs. intestinal (*n*, 5)	20	4
Haller et al., 2010 [32]	microarray	734	Gastric vs. intestinal (total *n*, 12)	4	7
Gits et al., 2013 [48]	microarray	725	GIST (*n*, 50) vs. leiomyosarcomas (*n*, 10)	7	14
Yamamoto et al., 2013 [40]	microarray	904	High risk (*n*, 10) vs. low risk (*n*, 4)	-	24
Kelly et al., 2013 [33]	RT-PCR	667	pediatric (*n*, 18) vs. adult GIST (*n*, 55)	30	10
Bachet et al., 2013 [46]	microarray	384	Cell lines (WT, D6, D54, WT/D6 and WT/D54); GIST samples WT (*n*, 3), PDGFRA mutations (*n*, 6) or KIT mutations (*n*, 11)	Different clustering of miRNAs based on mutation profile	
Akçakaya et al., 2014 [35]	microaaray	903	metastatic (*n*, 20) vs. non-metastatic (*n*, 10)	19	25
Tong et al., 2015 [36]	microarray	849	benign (*n*, 9) vs. malignant (*n*, 30)	3	1
Pantaleo et al., 2016 [45]	microarray	723	KIT/PDGFRA mutant (*n*, 4) vs. KIT/PDGFRA WT-SDH deficient GIST (*n*, 9)	16	40
Gyvyte et al., 2017 [47]	NGS	1672	GIST (*n*, 15) vs. GISTadjacent tissue (*n*, 15)	34	66

GIST—gastrointestinal stromal tumor, KIT—tyrosine kinase family, miRNA—microRNA, PDGFRA—platelet derived growth factor, SDH—succinate dehydrogenase, WT—wild type.

**Table 2:** Deregulated miRNAs and their target genes in GIST.

miRNA	Deregulation Pattern	Target Genes	Biological Effect	Reference
miR-17	downregulated	ETV1	inhibited cell proliferation, induced apoptosis	Gits et al., 2013 [48]
miR-20a	downregulated	ETV1	inhibited cell proliferation, induced apoptosis	Gits et al., 2013 [48]
miR-21	downregulated	BCL2	Aggravate the Imatinib-mediated growth inhibition and apoptosis	Cao et al., 2016 [50]
miR-34a	downregulated	PDGFRA	Suppressed cell proliferation	Isosaka et al., 2015 [37]
miR-125a	upregulated	PTPN18	Imatinib resistance	Akcakaya et al., 2016 [35]
miR-133b	downregulated	FSCN1	Enhanced proliferation	Yamamoto et al., 2013 [40]
miR-137	downregulated	TWIST1	inhibits cell migration	Liu et al., 2014 [51]
miR-152	downregulated	CTSL	inhibit proliferation, migration, invasion	Lu et al., 2017 [52]
miR-196a	upregulated	ANXA1	Invasion	Niinuma et al., 2012 [34]
miR-218	downregulated	KIT	inhibit proliferation and invasion	Fan et al., 2014 [38]
miR-221/222	downregulated	KIT, AKT, BCL2	inhibited cell proliferation, induced apoptosis	Ihle MA et al., 2015 [39] Koeltz M et al., 2011 [39] Gits et al., 2013 [53]
miR-494	downregulated	BIRC5, KIT	Suppressed proliferation; promote apoptosis and inhibite cell growth	Yun S et al., 2017 [54] Kim WK et al., 2011 [55]
miR-518a	downregulated	PIK3C2A	reduce proliferation and promote apoptosis	Shi Y et al., 2016 [43]

ANXA1—annexin A1; BCL2—B-cell lymphoma 2, BIRC5—Baculoviral IAP repeat-containing protein 5, CTSL—cathepsin L, ETV1—ets variant 1, FSCN-1—fascin actin-bundling protein 1, GIST—gastrointestinal stromal tumor, KIT—tyrosine kinase family, miRNA—microRNA, PDGFRA—platelet derived growth factor, PIK3C2A—Phosphatidylinositol-4-phosphate 3-kinase C2 domain-containing alpha polypeptide, PTPN18—protein tyrosine phosphatase non-receptor type 18, SDH—succinate dehydrogenase, TWIST1—twist family bHLH transcription factor 1, WT—wild type.

## Abbreviations

ANXA1	annexin A1
BCL-2	B-cell lymphoma 2
BIRC5	baculoviral IAP repeat-containing protein 5
CTSL	cathepsin L
ERK	extracellular signal-regulated kinase
ETV1	ets variant 1
FSCN-1	fascin actin-bundling protein 1
GIST	gastrointestinal stromal tumor
GRB2	growth factor receptor bound protein 2
isomiRs	isoforms of miRNAs
KIT	tyrosine kinase family
MDM2	murine double minute 2
microRNA	miRNA
MEK	mitogen-activated protein kinase
p53	tumor protein p53
PDGFRA	platelet derived growth factor
PIK3C2A	phosphatidylinositol-4-phosphate 3-kinase C2 domain-containing alpha polypeptide
PTPN18	protein tyrosine phosphatase non-receptor type 18
SDH	succinate dehydrogenase
SHC	squalene-hopene cyclase
SOS	son of sevenless gene
TWIST1	twist family bHLH transcription factor 1
WT	wild-type
